# Endoscopy-based IBD identification by a quantized deep learning pipeline

**DOI:** 10.1186/s13040-023-00350-0

**Published:** 2023-11-25

**Authors:** Massimiliano Datres, Elisa Paolazzi, Marco Chierici, Matteo Pozzi, Antonio Colangelo, Marcello Dorian Donzella, Giuseppe Jurman

**Affiliations:** 1 Fondazione Bruno Kessler, via Sommarive, 18, Trento, I-38123 Italy; 2 University of Trento, via Calepina, 14, Trento, I-38122 Italy; 3 GPI S.p.A., via Ragazzi del ’99, 13, Trento, I-38123 Italy

**Keywords:** Per-patient model, Interpretability, Quantization, Automatic preprocessing, Inflammatory bowel disease

## Abstract

**Background:**

Discrimination between patients affected by inflammatory bowel diseases and healthy controls on the basis of endoscopic imaging is an challenging problem for machine learning models. Such task is used here as the testbed for a novel deep learning classification pipeline, powered by a set of solutions enhancing characterising elements such as reproducibility, interpretability, reduced computational workload, bias-free modeling and careful image preprocessing.

**Results:**

First, an automatic preprocessing procedure is devised, aimed to remove artifacts from clinical data, feeding then the resulting images to an aggregated per-patient model to mimic the clinicians decision process. The predictions are based on multiple snapshots obtained through resampling, reducing the risk of misleading outcomes by removing the low confidence predictions. Each patient’s outcome is explained by returning the images the prediction is based upon, supporting clinicians in verifying diagnoses without the need for evaluating the full set of endoscopic images. As a major theoretical contribution, quantization is employed to reduce the complexity and the computational cost of the model, allowing its deployment on small power devices with an almost negligible 3% performance degradation. Such quantization procedure holds relevance not only in the context of per-patient models but also for assessing its feasibility in providing real-time support to clinicians even in low-resources environments. The pipeline is demonstrated on a private dataset of endoscopic images of 758 IBD patients and 601 healthy controls, achieving Matthews Correlation Coefficient 0.9 as top performance on test set.

**Conclusion:**

We highlighted how a comprehensive pre-processing pipeline plays a crucial role in identifying and removing artifacts from data, solving one of the principal challenges encountered when working with clinical data. Furthermore, we constructively showed how it is possible to emulate clinicians decision process and how it offers significant advantages, particularly in terms of explainability and trust within the healthcare context. Last but not least, we proved that quantization can be a useful tool to reduce the time and resources consumption with an acceptable degradation of the model performs. The quantization study proposed in this work points up the potential development of real-time quantized algorithms as valuable tools to support clinicians during endoscopy procedures.

## Background

Inflammatory Bowel Disease (IBD), including Crohn’s disease and Ulcerative Colitis, includes a group of chronic inflammatory disorders that affect the digestive tract. The main symptoms associated with IBD are persistent diarrhea, abdominal pain, rectal bleeding, weight loss, fever, anemia, anxiety and depression, and sometimes the condition also affects mouth or skin. Unfortunately, the incidence and prevalence of IBD is increasing globally [3, 16, 25], reducing the quality of life of millions of people [32]. Moreover, after the acute phase, patients may experience flare-ups followed by asymptomatic periods^1^. If these inflammations are not controlled, over time IBD can damage the intestine causing some severe problems like abscesses or increasing the colon cancer risk. Then, it is fundamental to diagnose IBD as soon as possible both to alleviate the symptoms and to avoid the appearance of other severe pathologies. IBD is diagnosed using endoscopy, radiology, histology or other bioimaging studies, such as MRI or computed tomography [13, 24]. Still, a precise and clearly defined criterion for diagnosing IBD is not available [31] and this uncertainty may frequently lead to misclassification or repeated examinations. More recently, several Artificial Intelligence (AI) applications have been developed, aimed to automatically discriminate whether a patient is affected by IBD or not [7, 9, 30]. On the same line, Takenaka and coauthors [29] proposed a convolutional neural network using endoscopic images as inputs. All these works implement a per-image (or imagewise) analysis, which means that the model’s input is an image and not the whole sequence of images associated to a patient, thus injecting an unwanted overoptimistic overfitting effect known as information (or data) leakage [6]. In the current proposal, our aims are to:

1. Introduce a preprocessing pipeline that can be used to improve the quality of the data in order to efficiently feed it to the algorithm and make the whole procedure more robust. In particular, clinical data may contain features that are operator or instrument dependent like black margins or external medical objects that can be misleading for the model;
2. Devise a per-patient model mimicking the doctor decision process by considering ensemble of the multiple predictions instead of multiple single image predictions. This helps the model to avoid misleading outcomes and return the sequence of snapshots that influence the model’s prediction with the corresponding confidence, thus introducing an explainability component within the model. Hence, this approach is more suitable for clinical applications.
3. Investigate whether quantization, i.e. a discretization procedure in the learning phase, can be used to optimize the model inference preserving the prediction performance. This is essential for the deployment of this algorithm inside hospitals where the amount of computational resources is limited, or even for its implementation as a point-of-care test. Indeed, for a single patient thousand of pictures are routinely taken and consequently, the prediction time might experience a significant increase.

In what follows, all the ingredients of the introduced pipelines are first discussed; then results obtained with and without the quantization procedure are presented and discussed, concluding the manuscript with some final remarks.

## Methods

### The pipeline

**Fig. 1:**
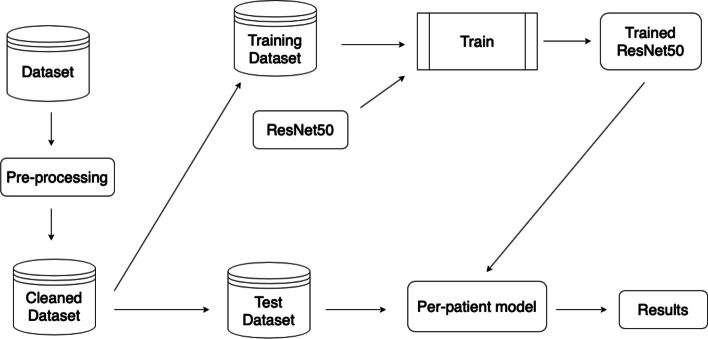
Overview of the methodology

The first component of the proposed workflow is an *ad-hoc* pre-processing pipeline aimed at cleaning the data, followed by a novel explainability feature embedded into the decision-making process. This aspect is of significant value from a clinical perspective as it enhances the transparency and interpretability of the model’s decisions. A general overview is presented in Fig. 1. As a first application, we used a subset of the SI-CURA dataset introduced in [9], focusing on discriminating 758 IBD patients from 601 healthy controls. The dataset has a patient-centric structure, *i.e.*, each patient is represented by a separate set of endoscopic images. After performing image pre-processing, we ensured that the patient-centric structure of the data remained invariant. Subsequently, we partitioned the dataset such that 80% of the patients were used for training, and the remaining 20% were used for testing. To train the model, we unpacked the training set such that each patient’s image was treated as an individual sample. In detail, a pretrained ResNet50 model is fine-tuned on the training dataset in a 5-fold cross validation schema to warrant reproducibility. The obtained trained model is then used to to construct an inference model that predicts the patient’s condition given his endoscopic images. Finally, the per-patient model is tested and evaluated on the test dataset.

### Data preprocessing phase

**Fig. 2:**
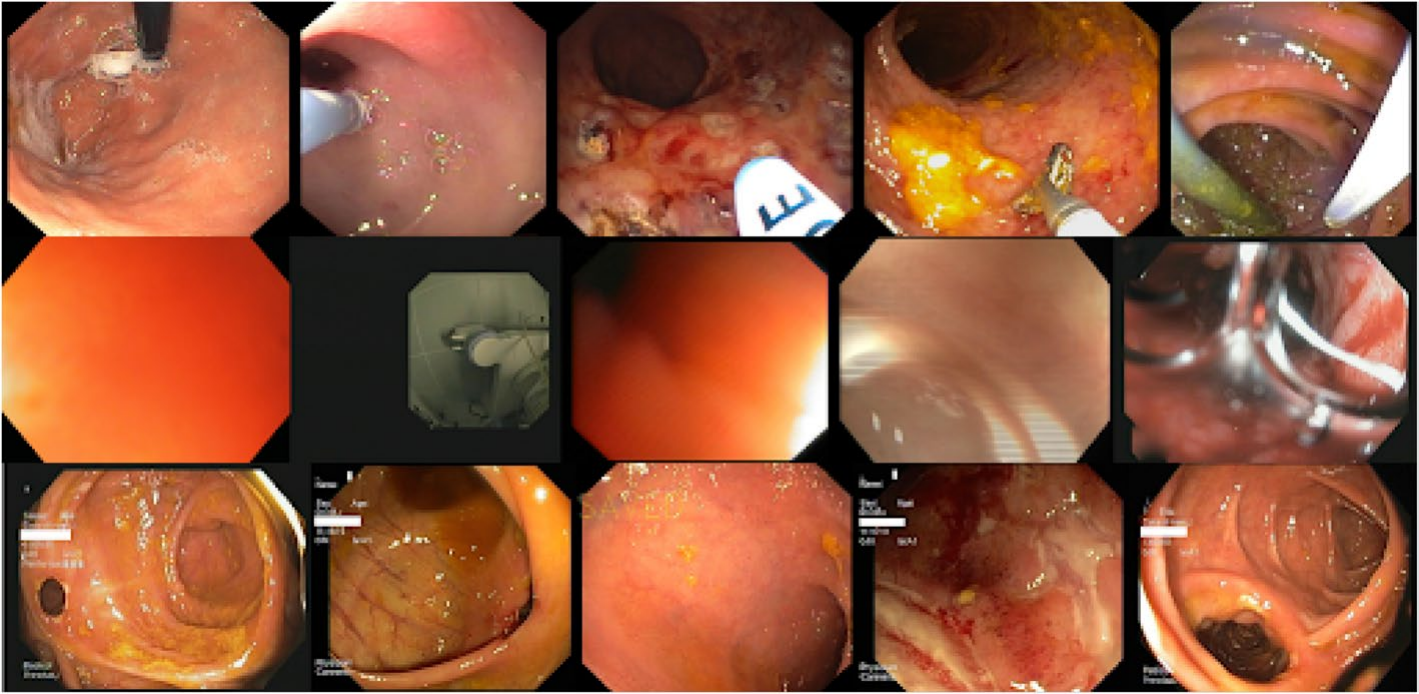
Samples containing artifacts and acquisition defects

**Fig. 3:**
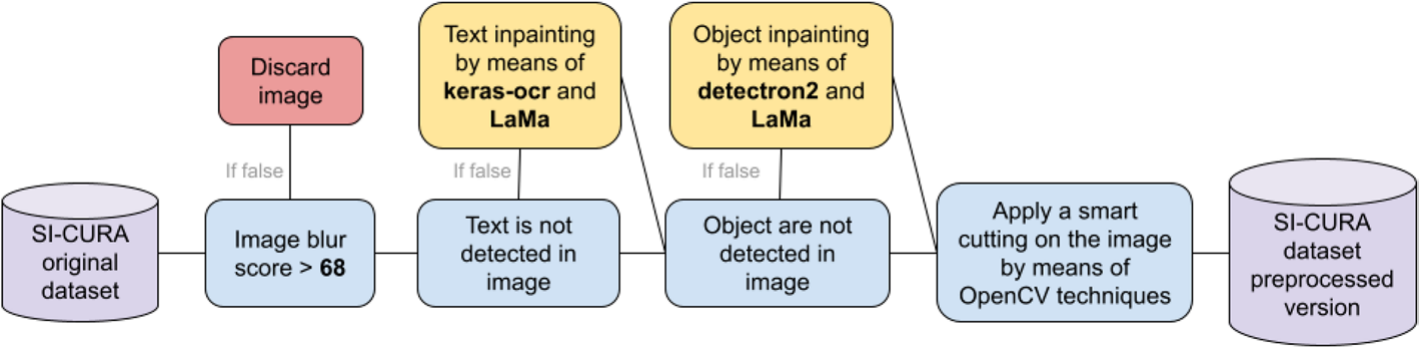
Detailed data preprocessing pipeline workflow

In the clinical domain, artifacts and acquisition problems are regularly affecting biomaging data, as shown by the examples reported in Fig. 2, resulting in a non negligible impact in terms of model effectiveness. The SI-CURA dataset contains data coming from different hospitals, making them heterogeneous and strongly dependent on the acquisition process and machines. In particular, raw data includes some completely uninformative images such as fully reflected or solid-colour pictures. Further, each image in this dataset is bordered by a black margin and its shape and size are different across different data; finally, the presence of text portions and visible medical artifacts like probes are frequently encountered. These aforementioned data issues were also highlighted during a brief interpretability analysis by means of Captum [19] methods such as Saliency [18, 26], Guided Backpropagation [22] and Occlusion [19], which provide some more insight regarding the areas and the patterns deemed as relevant at the prediction time. This analysis highlights how external objects such as text and medical tools turn out to be discriminating features for the classification task. To address this problem, we present a pre-processing pipeline capable of cleaning and harmonising data in an automatic and general context. A detailed overview of this pipeline is presented in Fig. 3.

In this respect, the implemented data preprocessing pipeline consists of the following steps: (1) apply the OpenCV Laplacian operator [2, 5] to assign a quality score to each image discarding images with score below a certain threshold; (2) for each image remove the text portion detected using Keras OCR^2^; (3) for each image fill the removed portion using in-painting algorithm LaMa network [28] which uses Fourier convolutions to fill in the deleted parts in a visually plausible and semantically coherent manner; (4) remove all the external object localizing them using MetaAI Detectron2 model [34] trained on a manually annotated subsection of the SI-CURA dataset and restore the removed part through LaMa network; (5) crop and resize each image to remove all the black margins. Note that this pipeline is quite general except for (4) and it does not require human supervision. Indeed, in case of data integration it is possible to preprocess data without any effort. Although it is not a perfect solution fitting all possible scenarios, this data preprocessing phase, designed to be integrated into the SI-CURA experimental workflow, is capable to addressed topics and issues as yet little discussed in the field of medical data, in which there are still no shared established techniques and methods for processing and cleaning steps.

### Model training

For our task, we opted to fine tune a ResNet50 model that was pre-trained on the ImageNet dataset, using the PyTorch Hub. In particular, we trained the last two convolutional layers and the last fully connected layer. To reduce the data splitting dependence, ensure robustness and prevent overfitting, we performed a 5-fold cross validation with Adam [17] as optimizer with a starting learning rate of 0.001. Decreasing the learning rate is a well known technique to improve both the trainability and the generalization properties of a model: hence, we decreased the learning rate by a factor of 0.1 every 20 epochs. This approach can help the model converge more effectively and fine-tune the model’s performance. Initially using a higher learning rate allows for larger updates to the model’s parameters, while decreasing it over time allows for smaller adjustments, which can lead to better convergence and improved generalization. Moreover, we used LASSO (Least Absolute Shrinkage and Selection Operator) regularization which encourages the model to have sparse weight values by adding a penalty term to the loss function. This prevents the model from becoming too complex and overfitting the training data, by encouraging the model to prioritize fewer important features and reduce the impact of less relevant features.

### Per-patient inference model

To enhance the model’s interpretability and mimic the clinician’s decision process, we build a per-patient model. During an endoscopy, clinicians perform a diagnostic examination of the interior of patient’s body using an endoscope, which is a flexible tube with a light and camera attached to it. The camera captures real-time snapshots of the internal organs or structures and doctors visually examines these images on a monitor, looking for any abnormalities, lesions, tumors, inflammation, bleeding, or other relevant findings. Therefore, at the end of the exam, for each patient a sequence of snapshots are available and the length of this sequence varies in a patient-specific manner, meaning it can differ from patient to patient. Hence the model’s inputs are 4-dimensional tensors whose slices are the RGB image corresponding to the snapshots aforementioned. Each image is then classified by the ResNet50 model independently producing a sequence of outcomes with the corresponding probabilities. The model’s results are subsequently sorted based on their confidence levels, and predictions with confidence below a threshold value, *t*, are discarded. The remaining predictions are then ensembled through a top-*k* weighted mean. This approach involves selecting the *k* highest confident predictions and then calculate a weighted mean where the weights are given by the confidence itself. During the fine-tuning of the hyperparameters, if all the snapshots of a certain patient are discarded for a certain *t* the models returns “Unpredicted” and the prediction is considered wrong. If all the model predictions falls below the threshold *t*, it means that the model confidence is too low for that patient and it does not have enough certainty to extract a reliable decision. The higher the threshold *t*, the less the model influences the doctor’s opinion, and the best possible choice of *t* is the highest one that both preserves the performance metrics and allows the model to be helpful in the majority of the cases. This helps to avoid the dissemination of misleading information where the model’s confidence is too low. The *k* hyperparameter allows the model to include not only the most confident prediction, but one which represents a more robust and reliable approach. Indeed, data can be noisy or encounter distribution shifts [14]. This induces models to produce high confident incorrect predictions, that can be leveraged by ensembling more than one single patient’s image prediction. Both *t* and *k* are chosen through a grid search with $t\in \{0.85, 0.87, 0.88, 0.90, 0.91, 0.93, 0.94, 0.96, 0.97, 0.99\}$ and $k\in \{1,2,3,4,5,6,7,8,9,10\}$. We select the tuple (*t*, *k*) that maximizes the Matthews Correlation Coefficient (MCC) [8, 15]. The grid search results are reported in Tables 1, 2, and 3. It is worth noticing that different combinations of *t* and *k* yield the same results, meaning that the model is able to identify the presence of IBD with high confidence and the per-image predictions are consistent within the same patient. Anyway, to minimize the dissemination of misleading information and to include in the prediction as much information as possible for each sample, the choice of the optimal (*t*, *k*) tuple was guided by maximizing the MCC metric. The choice of MCC is recommended since it is a robust statistical measure that assigns a high score only when the prediction achieves good results across all four categories of the confusion matrix proportionally on both the positive and negative cases in the dataset [8]. According to this criteria, the chosen tuple is $(t,k) = (0.93, 6)$ and in this case it maximizes also the accuracy and the $F_1$-score. Furthermore, we have tested the frequency of the “Unpredicted” samples varying the confidence threshold (see Table 4). Notice that $t=0.93$ is the highest threshold that guarantees a prediction for all validation samples. Another point in favor of the patientwise model’s reliability is the structure of the model’s outcomes. Indeed, in addiction to the patient’s health state prediction, clinicians can visually check the snapshots that influence the model’s outcome. This is an important features in clinical applications since it enables to perform a quick and easy verification.

**Table 1:** Grid search results on different values of the couple (*t*, *k*) where *t* is the threshold and *k* mean parameter when accuracy serves as metric

Per-Patient model Accuracy										
t$\setminus$k	1	2	3	4	5	6	7	8	9	10
0.85	0.977	0.977	0.977	0.977	0.977	0.977	0.972	0.972	0.967	0.958
0.87	0.972	0.972	0.972	0.972	0.972	0.972	0.967	0.967	0.963	0.953
0.88	0.977	0.977	0.977	0.977	0.977	0.977	0.972	0.972	0.967	0.958
0.90	0.977	0.977	0.977	0.977	0.977	0.977	0.972	0.972	0.967	0.958
0.91	0.977	0.977	0.977	0.977	0.977	0.977	0.972	0.972	0.967	0.958
0.93	0.977	0.977	0.977	0.977	0.977	0.977	0.972	0.972	0.967	0.958
0.94	0.972	0.972	0.972	0.972	0.972	0.972	0.967	0.967	0.963	0.958
0.96	0.972	0.972	0.972	0.972	0.972	0.972	0.967	0.967	0.963	0.958
0.97	0.972	0.972	0.972	0.972	0.972	0.972	0.967	0.967	0.963	0.958
0.99	0.972	0.972	0.972	0.972	0.972	0.972	0.967	0.967	0.963	0.958

**Table 2:** Grid search results on different values of the couple (*t*, *k*) where *t* is the threshold and *k* mean parameter when MCC serves as metric

Per-Patient model MCC										
t$\setminus$k	1	2	3	4	5	6	7	8	9	10
0.85	0.954	0.954	0.954	0.954	0.954	0.954	0.945	0.945	0.935	0.916
0.87	0.945	0.945	0.945	0.945	0.945	0.945	0.936	0.936	0.926	0.910
0.88	0.954	0.954	0.954	0.954	0.954	0.954	0.945	0.945	0.935	0.916
0.90	0.954	0.954	0.954	0.954	0.954	0.954	0.945	0.945	0.935	0.916
0.91	0.954	0.954	0.954	0.954	0.954	0.954	0.945	0.945	0.935	0.916
0.93	0.954	0.954	0.954	0.954	0.954	0.954	0.945	0.945	0.935	0.916
0.94	0.945	0.945	0.945	0.945	0.945	0.945	0.936	0.936	0.926	0.916
0.96	0.945	0.945	0.945	0.945	0.945	0.945	0.936	0.936	0.926	0.916
0.97	0.945	0.945	0.945	0.945	0.945	0.945	0.936	0.936	0.926	0.916
0.99	0.945	0.945	0.945	0.945	0.945	0.945	0.936	0.936	0.926	0.916

**Table 3:** Grid search results on different values of the couple (*t*, *k*) where *t* is the threshold and *k* mean parameter when $F_1$-score is used as metric

Per-Patient model $F_1$-score										
t$\setminus$k	1	2	3	4	5	6	7	8	9	10
0.85	0.979	0.979	0.979	0.979	0.979	0.979	0.974	0.974	0.970	0.962
0.87	0.974	0.974	0.974	0.974	0.974	0.974	0.970	0.970	0.966	0.958
0.88	0.979	0.979	0.979	0.979	0.979	0.979	0.974	0.974	0.970	0.962
0.90	0.979	0.979	0.979	0.979	0.979	0.979	0.974	0.974	0.970	0.962
0.91	0.979	0.979	0.979	0.979	0.979	0.979	0.974	0.974	0.970	0.962
0.93	0.979	0.979	0.979	0.979	0.979	0.979	0.974	0.974	0.970	0.962
0.94	0.974	0.974	0.974	0.974	0.974	0.974	0.970	0.970	0.966	0.962
0.96	0.974	0.974	0.974	0.974	0.974	0.974	0.970	0.970	0.966	0.962
0.97	0.974	0.974	0.974	0.974	0.974	0.974	0.970	0.970	0.966	0.962
0.99	0.974	0.974	0.974	0.974	0.974	0.974	0.970	0.970	0.966	0.962

**Table 4:** Number of validation patients that the per-patient model can not classify since the prediction confidence is too small for that specific value of the threshold *t*

Threshold (t)	Unpredicted
0.85	0
0.87	0
0.88	0
0.90	0
0.91	0
0.93	0
0.94	1
0.96	1
0.97	2
0.99	4

### Quantization

The patient-by-patient inference leads to a more robust patient prediction, albeit at the expense of longer inference time and increased resource consumption. Specifically, to predict a patient’s health condition, the inference phase is applied to the sequence of the available images for that particular patient. In this second part of the work, we investigate how quantization preserves the evaluation metrics on this specific task and we assess the gain in terms of prediction time. The idea of quantizing neural networks (QNN) has been introduced in the 1990s [12, 21] to make the hardware neural networks implementation easier. Recently, it turned out that quantizing neural network provides benefits also in term of size reduction of standard neural networks architecture, which are characterized by millions of parameters and hence they are very demanding in terms of storage memory, and energy consumption. Indeed, this creates an obstacle towards their deployment on low-memory and low-power architectures or for fast inference constrained applications [23, 27]. The primary concept underlying quantization is to reduce the precision of both weights and activations without performance degradation. This is done by constraining the model’s parameters to live in a finite set and replacing the model’s activations with functions with finite range, such as linear combinations of Heaviside functions. One of the easiest and wider use technique to achieve quantization is the post training quantization (PTQ) [1, 11]. Fixed the quantization range $\mathcal {Q}$, the quantization is done through a quantizer function $Q:\mathbb {R} \rightarrow \mathcal {Q}$ that maps a real number *x* to $Q(x) = \text {round}\left( \frac{x}{s} + Z\right)$ where *s* and *Z* are the scale and the zero point respectively. The scale *s* and the zero *Z* play a central role in adjusting the magnitude of the input entries to be projected on $\mathcal {Q}$ in an optimal way. To quantize a multidimensional tensor, the function *Q* is applied element-wise setting $s = \frac{\beta - \alpha }{\beta _q - \alpha _q}$ and $Z = -\left( \frac{\alpha }{s} - \alpha _q\right)$ where $[\alpha , \beta ]$ is the clipping range of *x* and $[\alpha _q, \beta _q]$ are the smaller and the larger value in $\mathcal {Q}$, respectively. While $\alpha _q$ and $\beta _q$ are fixed *a priori*, we need some estimates for the interval $[\alpha , \beta ]$. In post training quantization the clipping range is estimated through the calibration process. During this procedure, the maximum and minimum element values are collected on a subset of the validation dataset by attaching particular modules after the specific modules we want to quantize. These statistics are then used to define $\alpha$ and $\beta$. There are mainly two different strategies to set the clipping range: the affine or asymmetric quantization scheme and the symmetric quantization scheme. The asymmetric quantization scheme [33] assigns the input range to the minimum and maximum observed values, that is $\alpha = \min (x)$ and $\beta = \max (x)$ and computes *s* and *Z* as explained above. The symmetric quantization scheme centers the input range around zero avoiding the needed of *Z* and sets $-\alpha = \beta = \max (|\max (x)|, |\min (r)|)$. The asymmetric quantization scheme is commonly employed to quantize the positive model activations, such as those produced by the ReLU function. On the other hand, the affine quantization scheme proves to be more suitable for quantizing weight tensors, which may include both positive and negative values. Quantization parameters can be computed whole tensor or separately for each channel. Clearly, the first approach enables a significant reduction in the required number of quantization parameters, and hence the model complexity, but it often ends up with poor performance. The channelwise approach performs better than the tensorwise approach, but its overall cost is slightly higher. To further improve the performance of QNNs, Quantization Aware Training (QAT) has been introduced [20, 33]. The fundamental idea behind this method is to simulate the quantization operation during the training phase to adapt the model to the final quantization. This simulated quantization, called also fake quantization, first performs a calibration though observers as in PQT to properly initialize the scale and zero point. Subsequently, all the quantization parameters are frozen, and the model undergoes training for several epochs. It is worth noting that during this stage, the observers serve not only as statistics collectors but they also perform a pseudo quantization. This implies that they apply the function *Q* to the respective tensors, yet the output is still treated as a continuous parameter. Note that training such kind of model is not trivial because the functions *Q* are piecewise constant. Hence, it is only piecewise differential and the gradients are zero almost everywhere. To address this issue, the Straight-through estimator (STE) has been proposed [4, 35]. This involves the biased estimation of the gradient as a hard threshold function, which means that the gradient of *x* is one if it belongs to the quantization range and zero otherwise. Another widely use quantization training algorithm is Parameterized clipping activation method (PACT) presented in [10]. The underlying PACT training scheme is the same as in QAT, but this time the scale parameter of the activation functions is considered as another trainable parameter. This is done by setting $s = \frac{\tilde{\alpha }}{\beta _q - \alpha _q}$ where $\tilde{\alpha }$ is the activation clipping parameter.

## Results and discussion

### Continuous model

First, results on the test set – obtained by the classical algorithm in floating point arithmetic – are shown in Table 5: mean $\mu$ of the three metrics are reported together with the radius $\rho$ of the 5%-95% Confidence Intervals (CI) obtained through $10^5$ bootstrap resampling, assuming normality in the metrics’ distribution.

**Table 5:** Model performance on the test set. MCC: Matthews Correlation Coefficient; Acc: accuracy; $F_1$: $F_1$-score; $\mu$ mean of the metric over $10^{5}$ bootstrap resampling; $\rho$ radius of the corresponding 5%-95% CI

Category	MCC		Acc		$F_1$	
	$\mu$	$\rho$	$\mu$	$\rho$	$\mu$	$\rho$
per-patient	0.90	$5\cdot 10^{-4}$	0.95	$2\cdot 10^{-4}$	0.96	$2\cdot 10^{-4}$
per-image	0.84	$2\cdot 10^{-4}$	0.94	$10^{-4}$	0.94	$7\cdot 10^{-5}$

### Quantized model

We present the results obtained by applying PTQ, QAT and PACT to our model in Table 6. We made attempts to quantize all layers of ResNet50, but unfortunately, this resulted in a significant degradation of the model’s performance. This phenomenon arises from the fact that layers with fewer parameters generally exhibit a larger quantization error, as their parameters hold crucial information for solving the specific task at hand. Consequently, they are highly involved in the final model output and even minor variations in their values can lead to a substantial decline in performance. Hence, we performed some trials to decide which layers should be quantized, seeking a trade off between minimizing the performance degradation and maximizing the quantization advantages. It turned out that keeping the first layer and the classifier unquantized allows achieving approximately 3% of the overall metrics reported in Table 5. The results for the different types of quantization are reported in Table 6 with the corresponding bootstrap CIs.

**Table 6:** Partially quantized model performance on the test set. MCC: Matthews Correlation Coefficient; Acc: accuracy; $F_1$: $F_1$-score; $\mu$ mean of the metric over $10^{5}$ bootstrap resampling; $\rho$ radius of the corresponding 5%-95% CI

Method	MCC		Acc		$F_1$		Unpredicted
	$\mu$	$\rho$	$\mu$	$\rho$	$\mu$	$\rho$	
PTQ	0.82	$6\cdot 10^{-4}$	0.91	$3\cdot 10^{-4}$	0.91	$4\cdot 10^{-4}$	2
PACT	0.86	$6\cdot 10^{-4}$	0.93	$3\cdot 10^{-4}$	0.93	$3\cdot 10^{-4}$	2
QPWL	0.87	$5\cdot 10^{-4}$	0.94	$3\cdot 10^{-4}$	0.93	$3\cdot 10^{-4}$	1
QAT	0.82	$6\cdot 10^{-4}$	0.91	$3\cdot 10^{-4}$	0.91	$3\cdot 10^{-4}$	1

Notice that PTQ is outperformed by the other two methods. In the table the best obtained results is reported, but the variance in performance degradation obtained with this method is high depending on the calibration set. Next, we explore the impact of quantization on the model’s weight and its effect on inference time when running on a CPU. The storage capacity to save a floating-point (FP) ResNet50 model amounts to 95.50 MB, whereas saving the quantized optimal configuration requires 24.52 MB, one-fourth of the storage consumption of the FP counterpart. We performed also a speed test on a 12th Gen Intel(R) Core(TM) i9-12900KF comparing the quantized per-patient against the FP per-patient inference speed. It turns out that the quantized model is twice as faster compared to FP computation, requiring 6.87 seconds compared to 14.37 seconds to predict the health condition of the same patient.

## Conclusion

In this study, we have developed and assessed a specific deep learning framework to automatically identify inflammatory bowel disease. We showed that one of the major problems encountered when dealing with clinical data is the presence of artifacts that could affect the final model performance. Therefore, we presented a pre-processing pipeline to automatically detect and delete those artifacts producing a cleaned and ready-to-use dataset. Furthermore – guided by the idea of mimicking the clinical decision process – we have developed a per-patient based model that makes patient’s diagnoses taking into account also of the confidence of different predictions. This reflects into a model cautiousness that can be adjusted either by maximizing the overall task performance or by the user allowing the model to abstain from predicting when uncertainty occurs. Moreover, we explored a way towards interpretable design that can help healthcare professionals both in diagnosing IBD and in sifting through the extensive volume of images generated during endoscopic examinations. This is achieved by offering to the clinicians not only the patient’s prediction but also a set of *k* images that the model prioritizes to reach its decision. We tested this model specifically on IBD but having in mind a more general clinical framework in which it can be applied. The per-patient model achieves a test set performance MCC=0.90 for the classification of healthy controls versus IBD patients. Finally, we showed how quantization can significantly reduce the memory and the computational resources required for predictions. This empirical study on quantization performance provides also positive insights on the feasibility of making real-time predictions during endoscopy helping clinicians to identify the pathological areas in the affected bowel.

## Data Availability

The data generated and analysed during the current study are not publicly available due to privacy restrictions and commercial confidentiality, however they can be made available in a de-identified manner upon reasonable request and an appropriate agreement, contacting the corresponding author.

## Footnotes

1 https://www.hopkinsmedicine.org/health/conditions-and-diseases/inflammatory-bowel-disease

2 https://keras-ocr.readthedocs.io/en/latest/index.html

## Code availability

Source code is available upon request.
